# Causal Mediation Analysis in Single Case Experimental Designs: Introduction to the Special Issue

**DOI:** 10.1177/01632787211073194

**Published:** 2022-02-03

**Authors:** Milica Miočević, Mariola Moeyaert, Axel Mayer, Amanda K. Montoya

**Affiliations:** 1 Department of Psychology, 5620 McGill University, Montreal, QC, Montreal, QC, Canada; 2 Educational and Counseling Psychology, 1084 University at Albany, Albany, NY, USA; 3 Psychological Methods and Evaluation, 9167 Bielefeld University, Bielefeld, Germany; 4 8783 Psychology Department at UCLA, University of California Los Angeles (UCLA), Los Angeles, CA, USA

**Keywords:** mediation analysis, SCEDs, causal inference, introduction article, special issue

## Abstract

This special issue of *Evaluation and the Health Professions* is dedicated to methods for causal mediation analysis in Single Case Experimental Designs (SCEDs). Mediation analysis is used to identify intermediate variables that transmit the effect of the independent variable on the outcome. Until recently, mediation analysis was mostly confined to between-subjects designs and panel studies with few exceptions. Consequently, most of the developments in causal mediation analysis have also been restricted to such designs. In applied health research, SCEDs have been used to evaluate total effects of treatments on outcomes of interest. Providing researchers with the methods for evaluating causal indirect effects for individual participants can lead to important improvements in diagnosis, treatment, and prevention. This special issue includes articles that describe advanced quantitative methods for testing mediators in SCEDs, propose and test approaches that allow for relaxing statistical assumptions that may not hold in real data, and illustrate mediation analysis for a single participant in real and simulated SCEDs data.

This special issue of *Evaluation and the Health Professions* focuses on causal mediation analysis in Single Case Experimental Designs (SCEDs), covering a range of topics from evaluations of new and existing methods to applications. Mediation analysis focuses on three primary effects: 1) the total effect, the effect of the independent variable on the outcome; 2) the indirect effect, the effect of the independent variable on the outcome *through* the mediator(s); and 3) the direct effect, the effect of the independent variable on the outcome *not* transmitted through the mediator(s). Mediation analysis is used to identify intermediate variables that transmit the effect of the independent variable on the outcome, and to quantify the magnitude and test the significance of the indirect effect (MacKinnon, 2008). Since the publication of the seminal paper by Baron and Kenny (1986), mediation analysis has been used in thousands of studies in the health, social, and behavioral sciences. For example, it has been the method of choice for identifying *mechanisms* through which an HIV/STD risk-reduction intervention increases the probability of using condoms (O'Leary et al., 2008), how health workers’ resilience affects well-being (Maffoni et al., 2021), and how physical health affects mental health (Ohrnberger et al., 2017).

Advances in (causal) mediation analysis have clarified the necessary assumptions and appropriate procedures for identifying causal direct and indirect effects. In particular, three key concepts in causal mediation analysis are relevant for the current special issue: temporal order, confounding variables, and latent variables. With regard to temporal order, it is crucial that the independent variable *X* precedes the mediator *M* which in turn precedes the outcome *Y* (e.g., MacKinnon et al., 2007; Mayer et al., 2014). This can best be achieved in longitudinal designs. With regard to confounding variables, it is important that there are no unmeasured confounders for the *X*–*M* and the *M*–*Y* relationships (Judd & Kenny, 1981). While certain designs such as randomization of *X* can ensure that the *X*–*M* relationship is unconfounded, researchers need to think carefully about potential confounders of the *M*–*Y* relationship, even in randomized experiments. More precise mathematical formulations of causality conditions have been termed sequential ignorability (Imai, Keele, & Tingley, 2010; VanderWeele & Vansteelandt, 2009) or unbiasedness (Mayer et al., 2014). The plausibility of some, but not all, of these causality conditions can be checked via sensitivity analysis or statistical tests (e.g., Cox et al., 2013; Imai, Keele, & Yamamoto, 2010; Mayer et al., 2014; VanderWeele, 2010). With regard to latent variables, it is important to consider reliability and construct validity of all variables involved in the analysis, including observed variables in the mediation model and (un)measured confounders (Gonzalez & MacKinnon, 2021; Sengewald et al., 2019). Measurement error can seriously bias the total, direct, and indirect effects in causal mediation analysis (e.g., Hoyle & Kenny, 1999).

Modern approaches to causal mediation analysis define the effects of interest at the individual level. For example, the general approach to causal mediation popularized by Imai et al. (2010) in psychology defines individual (natural) direct and indirect effects using nested counterfactuals. However, only the average or conditional effects are routinely estimated in common designs such as pretest–posttest-follow-up control group designs. SCEDs, which can be considered a special case of interrupted time series designs (e.g., Kratochwill et al., 2010; Shadish et al., 2002), allow for investigating mediational processes at the individual level.

One of the advantages of using SCEDs is that individual participants serve as their own controls (i.e., participants are repeatedly measured before, during and/or after the intervention). Therefore, an individual-specific intervention effect can be estimated without a matching comparison group. The most basic SCED design involves one baseline condition (A-phase) that is “interrupted” by one intervention condition (B-phase). The effectiveness of the intervention can be evaluated by comparing intervention data with baseline (control) data (i.e., AB comparison). In order to increase the internal and external validity in SCEDs, it has been recommended to replicate the AB design within individuals (i.e., ABAB reversal designs) and across individuals (i.e., replicated AB designs or multiple-baseline designs across participants). Numerous effect sizes have been developed to represent intervention effectiveness for AB comparisons in SCEDs (Jamshidi et al., 2021). These effect sizes include non-overlap indices (e.g., percent of non-overlapping data, Scruggs et al., 1987; improvement rate difference, Parker et al., 2009; non-overlap of all pairs, Parker & Vannest, 2009; Tau-U, Parker et al., 2011; baseline corrected Tau-U; Tarlow, 2017), regression-based effect sizes (e.g., Moeyaert et al., 2014), the standardized mean difference (Hedges et al., 2012), and the log-response ratio (Pustejovsky, 2018) and related statistics (e.g., percent of goal obtained, Ferron et al., 2020). The regression-based approach is most promising for answering (causal) mediation questions for SCEDs that involve AB comparisons (e.g., Loeys, 2022; current issue).

Almost all methods for (causal) mediation analysis estimate the indirect effect at the group level. There are a few exceptions (Judd et al., 2001; Montoya & Hayes, 2017; Vuorre & Bolger, 2018) which still require data collection for more than one individual. Proposed methods for estimating indirect effects for a single individual in an AB design do not explicitly describe the required assumptions for making causal inferences about the indirect effect (Miočević et al., 2020). At the time of writing, there is only one proposed method for *causal* mediation analysis in SCEDs focusing on ABAB designs (Josephy et al., 2015). Causal mediation analysis methods have yet to be developed and tested for other kinds of SCED types, and this special issue aims to showcase current state of the art approaches and encourage further methodological developments in this area. The articles in this special issue describe the use of existing time series models, propose methods when statistical assumptions are not met, and provide illustrations using real and simulated data.

## Time Series Models

SCEDs are essentially time series designs where single individuals are assessed at many discrete time points. For the designs considered in this special issue, at least the mediator and the outcome are measured repeatedly at the individual level. Most methods in this special issue focus on the AB design; however, some of the proposed methods could be used when the independent variable is also a time series. In fact, the method described in MacKinnon et al. (2022, this issue) requires that the independent variable be randomly assigned at each time point. When analyzing time series data, we can draw on a large amount of literature from econometrics, biometrics, psychometrics, and related fields. A key characteristic of time series data is autocorrelation between neighboring measurements or residuals (Ferron, 2002; Shadish & Sullivan, 2011). The most basic models are autoregressive models and moving average models (for an introduction to time series models, see e.g., Cryer & Chan, 2008). These models can then be extended to also account for trends or seasonal components. Modeling trends and autocorrelation is especially important for analyzing data from SCEDs (e.g., Barlow, 2009; Ferron, 2002) and will be discussed in several articles in this special issue (Langenberg et al., 2022; this issue; Loeys, 2022; this issue; Somer et al., 2022, this issue). In principle, multiple time series could be analyzed separately using piecewise regression analysis techniques. However, mediation models for SCEDs not only require the modeling of a single time series but simultaneous modeling of several time series that are interrelated. There are different statistical approaches that allow for doing this such as vector autoregressive models, structural equation models, and state-space models (see Chow et al., 2010, for a comparison). The latter two approaches also allow for incorporating latent variables via measurement models. Langenberg et al. (2022, this issue) discussed and empirically validated a method, based on the state-space modeling approach by Gu et al. (2014), that can be used to estimate direct and indirect effects. State-space modeling is a flexible technique that can be used to estimate lagged effects among repeated measurements of multiple variables while also taking autocorrelation into account. Both of these complexities need to be modeled in contexts such as causal mediation for SCEDs. The authors recommend using a combination of maximum likelihood and permutation procedures to estimate *p*-values and standard errors, and recommend including at least 40 observations in the baseline condition and 40 observations in the intervention condition. Relatedly, Loeys (2022, this issue) and Somer et al. (2022, this issue) specifically focused on how to deal with the issue of autocorrelation (assuming a first order autocorrelation) in time series data using regression modeling techniques. Loeys (2022, this issue) compared three approaches, namely (1) transforming correlated errors into an uncorrelated sequence (also called “whitening”), (2) the Newey–West standard errors correction (i.e., correction that can deal with autocorrelation and heteroscedasticity in error terms), and (3) Feasible Generalized Least Squares estimation. They found small differences between the approaches for a small number of total observations (i.e., 15). However, for a larger number of observations (i.e., 30 and 90), the Feasible Generalized Least Squares approach is recommended. Findings from Somer et al. (2022) with complete data also suggest that the Feasible Generalized Least Squares approach outperforms alternative methods for handling autocorrelation in SCEDs.

## Mediation in Single Case Experimental Designs When Statistical Assumptions are not Met

Existing methods for evaluating mediation in SCEDs rely on assumptions which may not always be met in real data collection contexts. Often researchers must deal with small samples, missing data, or violations of distribution assumptions. Multiple papers in this special issue evaluate methods to help address these types of situations which researchers may encounter in applied settings.

MacKinnon et al. (2022, this issue) propose a new method, the Randomization Permutation test for mediation, where *X* is randomly assigned at each time point, and *M* and *Y* are measured at the same measurement occasions. This new method extends the work of Edgington and Onghena (2007) from a single outcome to a single mediator model, which allows for the estimation of the indirect effect. This method randomly permutes residuals from the model, and when the autocorrelation is modeled appropriately, the power to detect large indirect effects is adequate with approximately 50 time points. Even though SCEDs have historically had fewer than 50 time points (Shadish & Sullivan, 2011), future studies can attain the required sample sizes for adequate power using real-time monitoring technology (Bentley et al., 2019).

While MacKinnon et al., (2022, this issue) only examine one method for handling autocorrelation, two papers in this current issue directly investigate methods for handling autocorrelation (Loeys, 2022; this issue; Somer et al., 2022, this issue). Somer et al. (2022, this issue) explore multiple methods for modeling autocorrelation in combination with missing data handling methods. The authors find that Feasible Generalized Least Squares and the autoregressive model yield estimates of the indirect effect with the best statistical properties, and recommend multiple imputation for handling missing data. In addition to guiding readers through important use considerations for each method, all papers in the special issue contain empirical examples.

## Future Directions for Causal Mediation Analysis Research in Single Case Experimental Designs

In August 2019, we held a workshop on Single Subject Causal Mediation Analysis at the Lorentz Center in Leiden, the Netherlands. The workshop was attended by approximately 40 researchers who specialize in causal inference, mediation analysis, SCEDs, and clinical psychology. Many of the teams of co-authors who contributed to this special issue started developing their ideas at the workshop in the randomly assigned interdisciplinary groups of 4–5 people. Less than 3 years later, we are publishing a special issue to disseminate novel insights that advance the methodological literature and applications of causal mediation analysis in SCEDs. The software and detailed example interpretations will allow applied researchers to conduct mediation analysis in several SCED types, ranging from AB designs to models with random assignment of the intervention at each measurement occasion.

However, it is beyond the scope of the current special issue to provide a comprehensive overview of mediation analysis across all possible SCEDs. There are other designs for which causal mediation analysis for a single participant has yet to be described and tested, for example, parallel and sequential mediation models and moderated mediation models. Furthermore, methods for combining the results of several SCEDs via meta-analysis (see e.g., articles in the special issue in Evidence-Based Communication Assessment and Intervention edited by Schlosser & Sigafoos, 2008) could be extended to causal mediation models. Thus, this special issue will hopefully serve as a catalyst for more interdisciplinary collaboration, the proliferation of applications of mediation analysis in the fields of evaluation and the health professions, and novel methods for causal mediation analysis in SCEDs.
